# Transcriptomic and phylogenetic analysis of *Culex pipiens quinquefasciatus* for three detoxification gene families

**DOI:** 10.1186/1471-2164-13-609

**Published:** 2012-11-10

**Authors:** Liangzhen Yan, Pengcheng Yang, Feng Jiang, Na Cui, Enbo Ma, Chuanling Qiao, Feng Cui

**Affiliations:** 1 State Key Laboratory of Integrated Management of Pest Insects & Rodents, Institute of Zoology, Chinese Academy of Sciences, Beijing 100101, China; 2 Research Institute of Applied Biology, Shanxi University, Taiyuan, Shanxi, 030006, China

**Keywords:** Carboxyl/cholinesterases, Cytochrome P450 monooxygenases, Glutathione S-transferases, Insecticide resistance, Gene expansion, Gene expression

## Abstract

**Background:**

The genomes of three major mosquito vectors of human diseases, *Anopheles gambiae*, *Aedes aegypti*, and *Culex pipiens quinquefasciatus*, have been previously sequenced. *C. p. quinquefasciatus* has the largest number of predicted protein-coding genes, which partially results from the expansion of three detoxification gene families: cytochrome P450 monooxygenases (P450), glutathione S-transferases (GST), and carboxyl/cholinesterases (CCE). However, unlike *An. gambiae* and *Ae. aegypti*, which have large amounts of gene expression data, *C. p. quinquefasciatus* has limited transcriptomic resources. Knowledge of complete gene expression information is very important for the exploration of the functions of genes involved in specific biological processes. In the present study, the three detoxification gene families of *C. p. quinquefasciatus* were analyzed for phylogenetic classification and compared with those of three other dipteran insects. Gene expression during various developmental stages and the differential expression responsible for parathion resistance were profiled using the digital gene expression (DGE) technique.

**Results:**

A total of 302 detoxification genes were found in *C. p. quinquefasciatus*, including 71 CCE, 196 P450, and 35 cytosolic GST genes. Compared with three other dipteran species, gene expansion in *Culex* mainly occurred in the CCE and P450 families, where the genes of α-esterases, juvenile hormone esterases, and CYP325 of the CYP4 subfamily showed the most pronounced expansion on the genome. For the five DGE libraries, 3.5-3.8 million raw tags were generated and mapped to 13314 reference genes. Among 302 detoxification genes, 225 (75%) were detected for expression in at least one DGE library. One fourth of the CCE and P450 genes were detected uniquely in one stage, indicating potential developmentally regulated expression. A total of 1511 genes showed different expression levels between a parathion-resistant and a susceptible strain. Fifteen detoxification genes, including 2 CCEs, 6 GSTs, and 7 P450s, were expressed at higher levels in the resistant strain.

**Conclusions:**

The results of the present study provide new insights into the functions and evolution of three detoxification gene families in mosquitoes and comprehensive transcriptomic resources for *C. p. quinquefasciatus*, which will facilitate the elucidation of molecular mechanisms underlying the different biological characteristics of the three major mosquito vectors.

## Background

Mosquitoes are the most important vectors of human diseases. The *Culex pipiens* complex has a broad geographic distribution and is the vector of the West Nile virus and the *Wuchereria bancrofti* nematode, which causes filariasis. Over the last several decades, chemical insecticides have been intensively applied to control disease transmission. However, such control is undermined seriously by the increased insecticide resistance of vector mosquitoes. Three gene families are implicated in insecticide metabolism in mosquitoes: cytochrome P450 monooxygenases (P450s) are responsible for pyrethroid resistance [1], glutathione S-transferases (GSTs) are responsible for DDT resistance [2], and carboxyl/cholinesterases (CCEs) are responsible for organophosphate and carbamate resistance [3]. Many insect species show rapid expansion and diversification of detoxification genes, as disclosed by their sequenced genomes. The expansion or restriction of detoxification genes likely helps insects adapt to their particular ecological niches and enable them to survive natural and man-made insecticide selection.

The genomes of three major taxonomic mosquitoes, including *Anopheles gambiae*, *Aedes aegypti*, and *Culex pipiens quinquefasciatus*, have been analyzed and released to the public [4-6]. Of the three, *Ae. aegypti* has the largest genome size (1376 Mb), while *C. p. quinquefasciatus* has the largest number of predicted protein-coding genes (18883), which is 22% larger than that of *Ae. aegypti* and 52% larger than that of *An. gambiae*. The extra number of protein-coding genes partially results from the expansion of its three detoxification gene families. However, unlike *An. gambiae* and *Ae. aegypti*, which have large amounts of gene expression data, such as various expressed sequence tag libraries and transcriptomes, *C. p. quinquefasciatus* has limited gene expression resources, with only several salivary gland transcriptomes currently reported [7,8]. Knowledge of complete gene expression information is very important for the exploration of the functions of genes involved in specific biological processes and for the discovery of new candidate genes.

In the present study, the three detoxification gene families of *C. p. quinquefasciatus* were subjected to phylogenetic analysis and compared with those of three other dipteran insects. The CCE and P450 families were found to undergo large gene expansion. Digital gene expression tag profiling (DGE) technology was used to perform a deep transcriptome analysis of *C. p. quinquefasciatus* during development and in response to organophosphate insecticide selection. The gene expression profiles obtained provide an invaluable resource for the identification of genes involved in the development and insecticide resistance of *C. p. quinquefasciatus*.

## Results and discussion

### *C. p. quinquefasciatus* detoxification gene families

When 1e^-10^ was used in the HMMER searches, 79, 203, and 17 candidate genes of CCEs, P450s, and GSTs were identified in the *C. p. quinquefasciatus* genome, respectively. After verified by community annotations, only 71 CCEs and 196 P450s were confirmed. The eight false positives for CCEs were lipases or conserved hypothetical proteins and the seven false positives for P450s were groucho protein, 25-hydroxyvitamin D-1 alpha hydroxylase, or conserved hypothetical proteins. When the search stringency was lessened to 2e^-2^, 35 cytosolic GSTs were identified and supported by community annotations. Thus, a total of 302 detoxification genes were found in *C. p. quinquefasciatus*, including 71 CCE, 196 P450, and 35 GST genes, representing the widest gene expansion among the dipteran insect genomes sequenced thus far (Table 1). Gene expansion was mainly observed in the CCE and P450 families compared with three other dipteran species.

**Table 1:** **Classification of detoxification gene families in *Drosophila melanogaster*, *Anopheles gambiae*, *Aedes aegypti*, and *Culex pipiens quinquefasciatus***

	***D. melanogaster***	***A. gambiae***	***A. aegypti***	***C. p. quinquefasciatus***
CCE				
Dietary/detoxification^*^				
B class (α-esterases)	13	16	22	30 (18)
Hormone/semiochemical processing				
D class (integument esterases)	3	0	0	1 (0)
E class (β-esterases)	2	4	2	3 (3)
F class (dipteran JH esterases)	3	6	7	13 (5)
G class (lepidopteran JH esterases)	0	4	6	9 (6)
Neuro/developmental				
H class (glutactins)	5	10	7	6 (5)
I class (unknown)	1	1	1	1 (1)
J class (acetylcholinesterases)	1	2	2	2 (2)
K class (gliotactins)	1	1	1	1 (1)
L class (neuroligins)	4	5	5	3 (3)
M class (neurotactins)	2	2	2	2 (2)
Total	35	51	55	71 (46)
P450				
CYP2	6	10	11	14 (12)
CYP3 (include CYP6 and CYP9)	36	42	84	88 (72)
CYP4	32	45	59	83 (58)
Mitochondrial	11	9	10	11 (8)
Total	85	106	164	196 (150)
GST				
Delta	11	12	8	14 (13)
Epsilon	14	8	8	10 (6)
Omega	5	1	1	1 (1)
Sigma	1	1	1	1 (1)
Theta	4	2	4	6 (6)
Zeta	2	1	1	0 (0)
Others	0	3	3	3 (2)
Total	37	28	26	35 (29)

*The dietary/detoxification functional group follows the new system proposed by Oakeshott et al. (2010).

Data of *D. melanogaster*, *An. gambiae*, and *Ae. aegypti* are taken from Oakeshott et al. (2010).

The number of *C. p. quinquefasciatus* genes in brackets is from the DGE libraries.

CCE, carboxyl/cholinesterases; P450, cytochrome P450 monooxygenases; GST, glutathione S-transferases.

**Figure 1:**
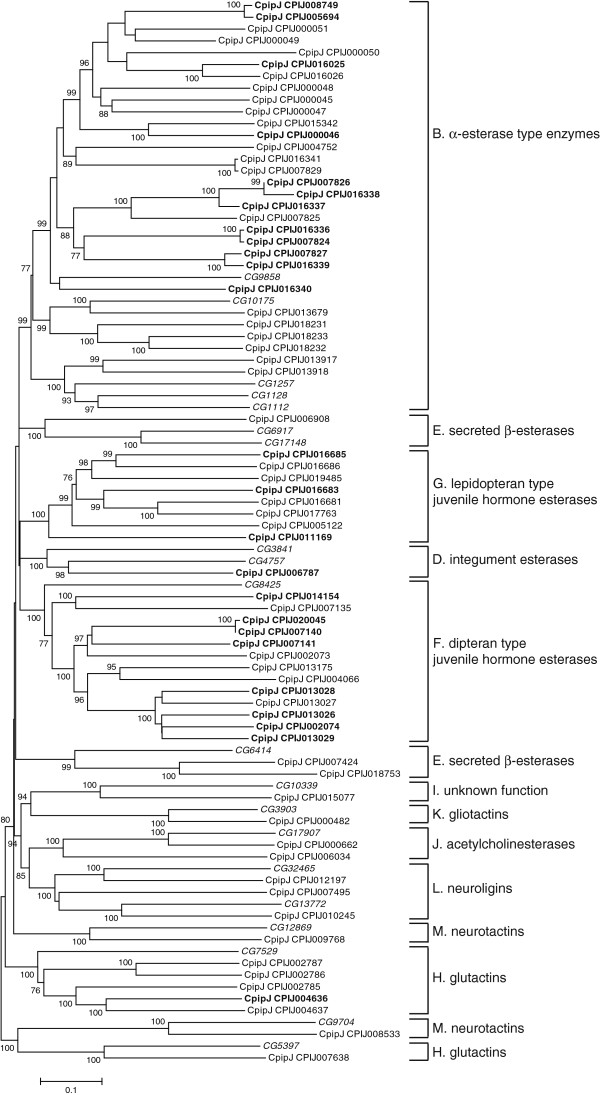
**Unrooted distance neighbor-joining tree showing the phylogeny of carboxyl/cholinesterases (CCEs) from the genome of *Culex pipiens quinquefasciatus *in relation to CCEs from *Drosophila melanogaster *(in italics).** CCEs of *C. p. quinquefasciatus* undetected in DGE libraries are shown in bold. The percentage of bootstrap confidence values greater than 70% (1000 replicates) is shown at the nodes. The functional assignment of clades follows the new system proposed by Oakeshott et al. [9].

Seventy-one CCE sequences were detected in the *C. p. quinquefasciatus* genome, which was approximately 39% and 29% gene-expanded compared with *An. gambiae* and *Ae. aegypti*, respectively, and 2-fold the number of CCEs found in *D. melanogaster* (Table 1, Additional file 1). The new functional assignment proposed by Oakeshott et al. [9] was used to designate the clades in the CCE phylogeny (Figure 1). A total of 11 clades, representing dietary/detoxification, hormone/semiochemical processing, and neuro/developmental functions, were obtained. The numbers of *C. p. quinquefasciatus* CCEs in the three functional classes were 30, 26, and 15, respectively (Table 1).

The number of CCEs in the neuro/developmental class was relatively conserved among the four dipteran insects. Similar conservation occurs in hymenopteran (*Nasonia vitripennis*, *A. mellifera*) and coleopteran (*Tribolium castaneum*) genomes [9], which reflects the relatively ancient origins of this class, where all members are catalytically inactive except for the acetylcholinesterases.

**Figure 2:**
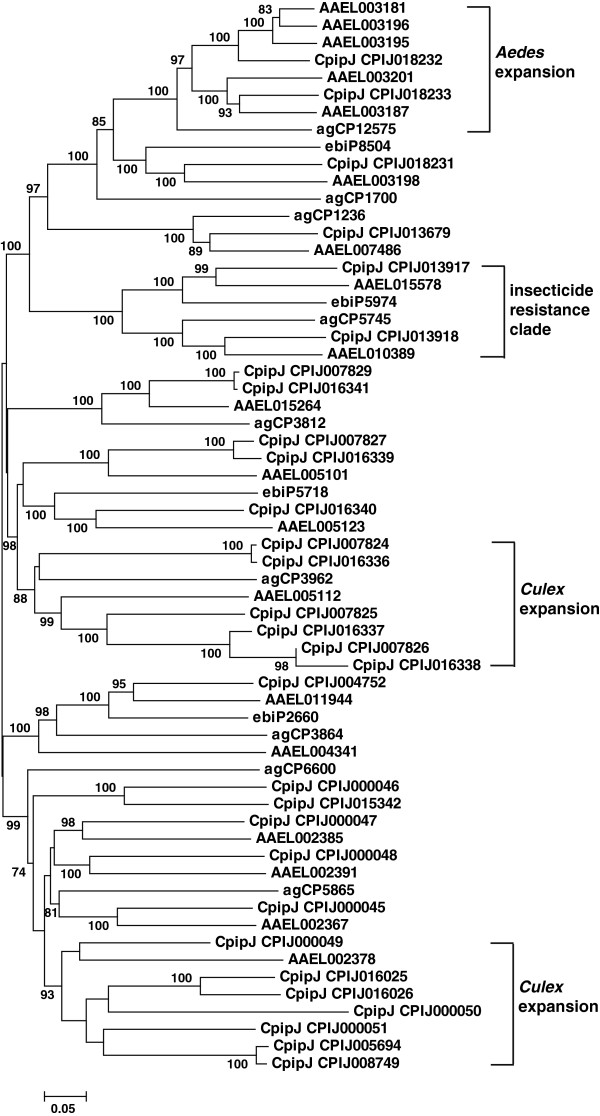
**Unrooted distance neighbor-joining tree showing the phylogeny of α-esterases from the genome of *Culex pipiens quinquefasciatus *in relation to those from *Aedes aegypti *(initiated with ‘AAEL’) and *Anopheles gambiae *(initiated with ‘agCP’ or ‘ebiP’).** The percentage of bootstrap confidence values greater than 70% (1000 replicates) is shown at the nodes.

CCEs in dietary/detoxification and hormone/semiochemical processing classes expanded on the *C. p. quinquefasciatus* genome compared with the three other dipteran insects. Expansion mainly occurred in α-esterases (30 genes in clade B) and juvenile hormone esterases (22 genes in clades F and G). For α-esterases *Culex* showed rapid radiation in two clusters, which contained six and seven members, respectively, and *Aedes* displayed an obvious expansion in one cluster with five α-esterases (Figure 2). The α-esterases are thought to be involved in the development of metabolic resistance to insecticides; some examples include αE7 of *Lucilia cuprina*[10] and esterase A (CpipJ_CPIJ013918) and esterase B (CpipJ_CPIJ013917) of *C. p. quinquefasciatus*[3]. Unlike the rapid radiation of other α-esterases, the esterase A and esterase B were well conserved: secure 1:1:1 orthologs were found across *Culex*, *Anopheles* and *Aedes* (Figure 2). Twenty-eight other α-esterases of the species are probably involved in the metabolism of endotoxins or naturally occurring dietary constituents. The α- and β-based nomenclatures are applied extensively to esterase isozymes in *Drosophila* according to their preferential hydrolysis of isomeric artificial substracts, α- and β-naphthyl acetate, respectively [11]. The nomenclatures in themselves represent no broad biological distinctions. In the culicines, two esterase genes involved in organophosphate resistance are commonly designated esterase A and esterase B based on their ability to use preferentially α- or β-naphthyl acetate in the presence of equal quantities of both substrates [3,12].

**Figure 3:**
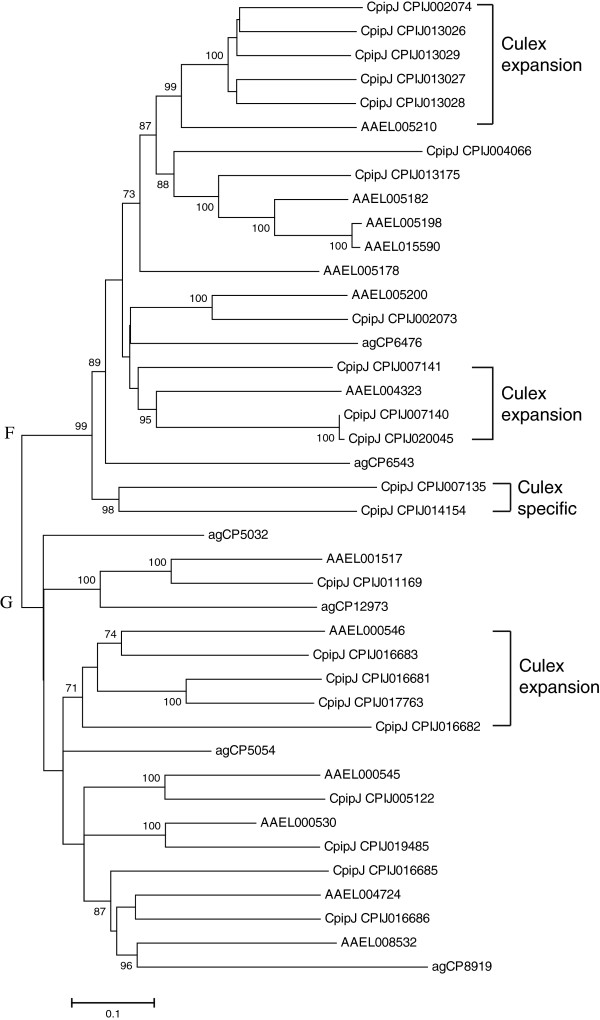
**Unrooted distance neighbor-joining tree showing the phylogeny of juvenile hormone esterases from the genome of *Culex pipiens quinquefasciatus *in relation to those from *Aedes aegypti *(initiated with ‘AAEL’) and *Anopheles gambiae *(initiated with ‘agCP’).** The percentage of bootstrap confidence values greater than 70% (1000 replicates) is shown at the nodes.

The large expansion of juvenile hormone esterases (JHEs) on the *C. p. quinquefasciatus* genome is interesting: 22 compared with 3 to 13 in three other dipteran genomes and 2 in the hymenopteran and coleopteran genomes [9]. When compared among the three mosquito species, the expansion in the *Culex* happened in three clusters, which included five, three, and four JHEs, respectively, and two JHEs were specific for the *Culex* (Figure 3). Among 22 JHEs, 10 have typical GQSAG nucleophilic elbow motifs around the catalytic site and 6 have varied motifs, such as GH (W/N/Y) SAG. Empirical functional data are required to determine the identity of juvenile hormone esterases.

The secreted β-esterases (clade E) are comparatively conserved among the three mosquito species: from 2 to 4 β-esterase genes were found in mosquito genomes, largely different from the expansion (11 β-esterase genes) found in *N. vitripennis* genome [9]. Some members of the β-esterases have well-described functions in other insects, such as E4 and FE4 esterases, which confer OP insecticide resistance in *Myzus persicae*[13], and the antennal Apo1PDE esterase of the silkworm *Antheraea polyphemus*, which degrades sex pheromones [14]. The functions of the β-esterases in *Culex* need to be further investigated.

**Figure 4:**
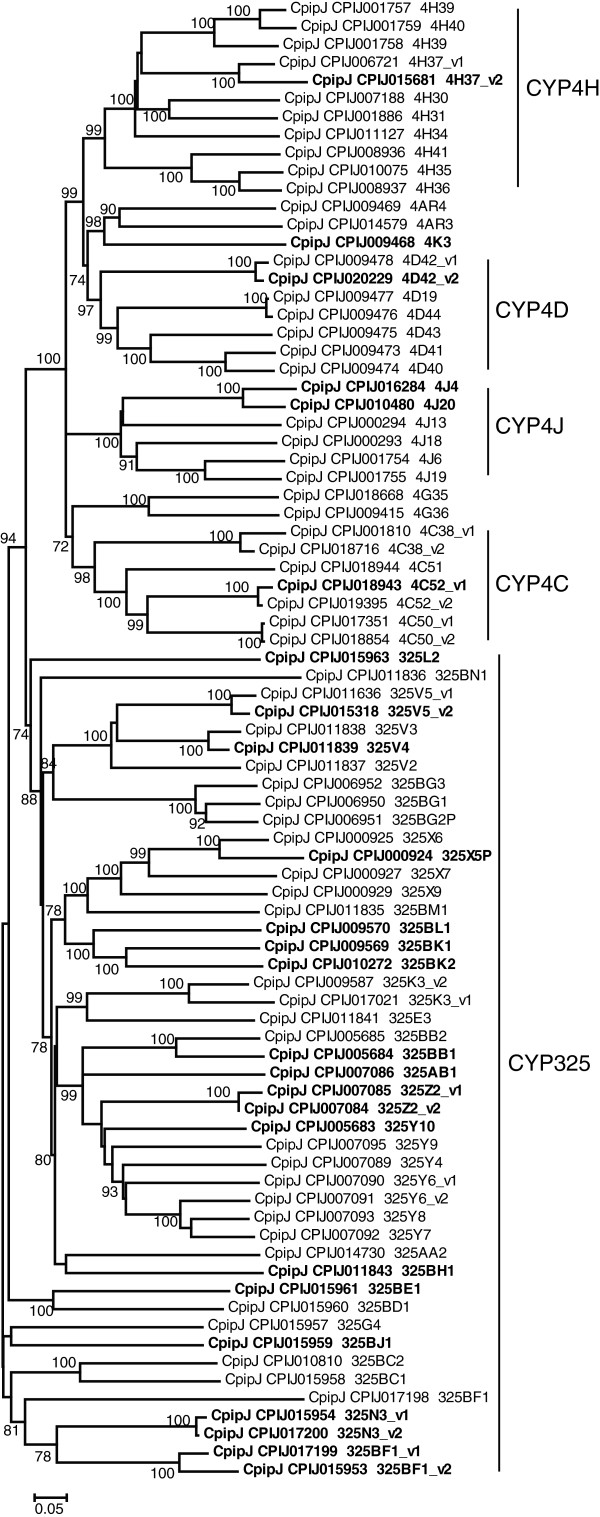
**Unrooted distance neighbor-joining tree of P450 CYP4 genes from the genome of *Culex pipiens quinquefasciatus*.** Greater than 70% support in 1000 bootstrap replications is indicated at the corresponding nodes. Genes undetected in DGE libraries are shown in bold. P450s were named by the P450 nomenclature committee ( http://drnelson.uthsc.edu/CytochromeP450.html). Genes with v1 and v2 designation are very recent duplications and have not yet been assigned individual gene names.

**Figure 5:**
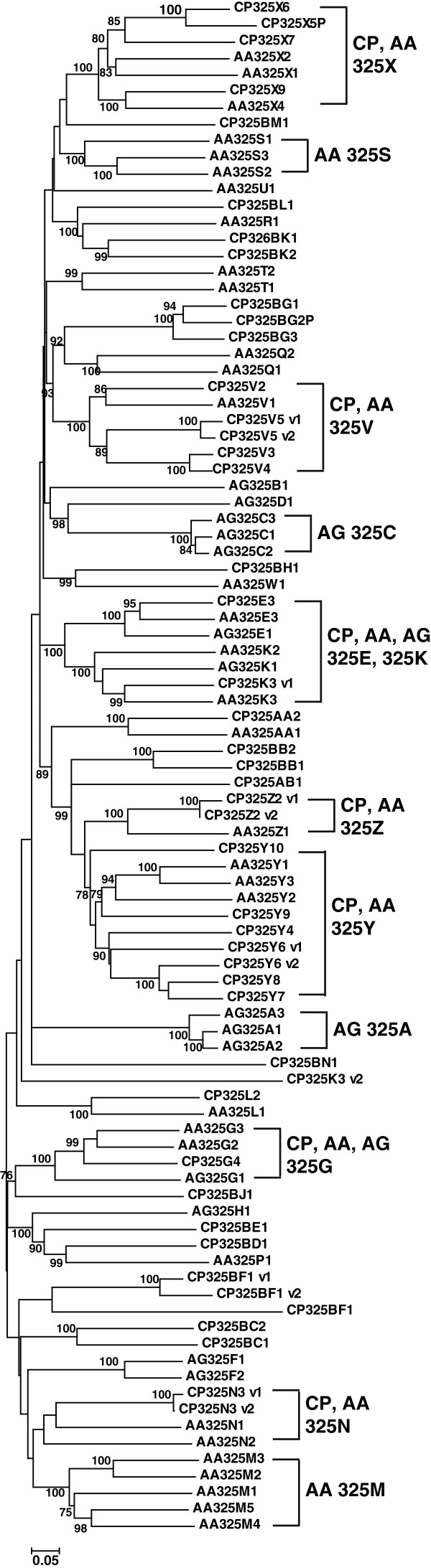
**Unrooted distance neighbor-joining tree showing the phylogeny of P450 CYP325 genes from the genome of *Culex pipiens quinquefasciatus *(initiated with ‘CP’) in relation to those from *Aedes aegypti *(initiated with ‘AA’) and *Anopheles gambiae *(initiated with ‘AG’).** The percentage of bootstrap confidence values greater than 70% (1000 replicates) is shown at the nodes.

Compared with the P450 genes of *D. melanogaster* and *An. gambiae*, those of *C. p. quinquefasciatus* expanded by 130% and 85%, respectively, and were slightly more than the number of those in *Ae. aegypti* (Table 1). Expansion was most pronounced in the CYP4 clade, where, among 83 CYP4 genes, 46 belonged to CYP325 (Figure 4). Some CYP325s were conserved among the three mosquito species, such as CYP325E, CYP325K, and CYP325G; some only expanded in the *Culex* and *Aedes* genomes, such as CYP325X, CYP325Y; even *Aedes* and *Anopheles* had their specific CYP325s (Figure 5). However, *Culex* is not evolved species-specific large CYP325 gene expansion. The physiological function of the CYP325 clade in insects remains unclear, except that a *CYP325A3* gene was found to be overexpressed in a permethrin-resistant strain of *An. gambiae*[15]. But this gene does not have clear orthologs in *Culex* or *Aedes* (Figure 5), indicating that the resistance mechanism of *CYP325A3* overexpression may be limited to *Anopheles*. Other large clades of CYP4 genes included CYP4H, CYP4D, CYP4J, and CYP4C (Figure 4), members of which are involved in DDT and pyrethroid insecticide resistance in mosquitoes [15,16]. The P450 genes were also expanded in *Ae. aegypti* compared to *An. gambiae*, but this expansion was most prominent in the CYP9 of CYP3 family: 37 CYP9 genes in *Ae. aegypti* contrasting to just 9 in *An. gambiae*[15].

**Figure 6:**
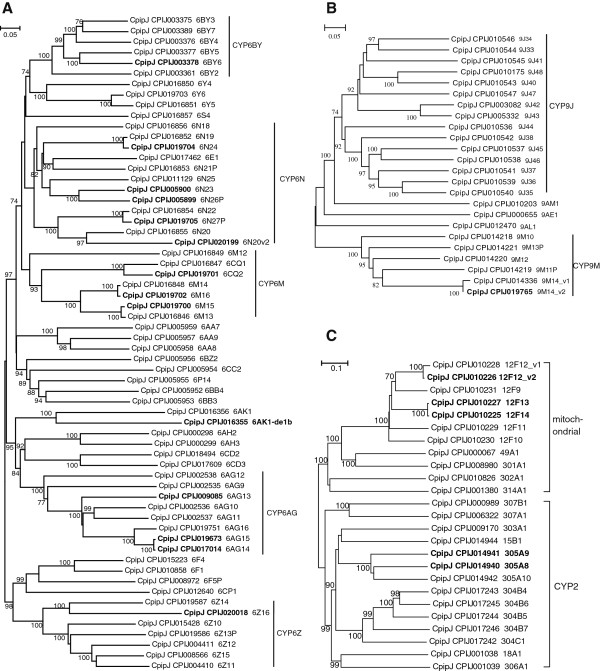
**Unrooted distance neighbor-joining tree of the P450 CYP6 (A), CYP9 (B), CYP2, and mitochondrial P450 genes (C) from the genome of *Culex pipiens quinquefasciatus*.** The presentation format is same as Figure 4.

While the total number of CYP3 members (including CYP6 and CYP9) in *C. p. quinquefasciatus* was similar to that in *Ae. aegypti*, *C. p. quinquefasciatus* had more CYP6 members and few CYP9 members than *Ae. aegypti*[17]. Sixty-three CYP6 genes of *C. p. quinquefasciatus* were mainly distributed in the CYP6BY, CYP6N, CYP6M, CYP6AG, and CYP6Z groups (Figure 6A), and 25 CYP9 genes were mainly distributed in the CYP9J and CYP9M groups (Figure 6B). Members of CYP6 have been implicated in resistance to a broad range of insecticides (e.g., OPs, pyrethroids, DDT, and neonicotinoids) in many insects [15,18-20], while less evidence implicates CYP9s in the detoxification of insecticides. In 11 mitochondrial P450s, 7 genes belonged to CYP12F (Figure 6C). Members of the CYP12 clade are involved in DDT resistance in *An. gambiae*[15] and *D. melanogaster*[21]. Several large clusters of P450 genes were found in the *C. p. quinquefasciatus* genome, such as a cluster of 13 CYP9 genes on supercontig 278 and a cluster of 12 CYP6 genes on supercontig 869 (Additional file 2).

**Figure 7:**
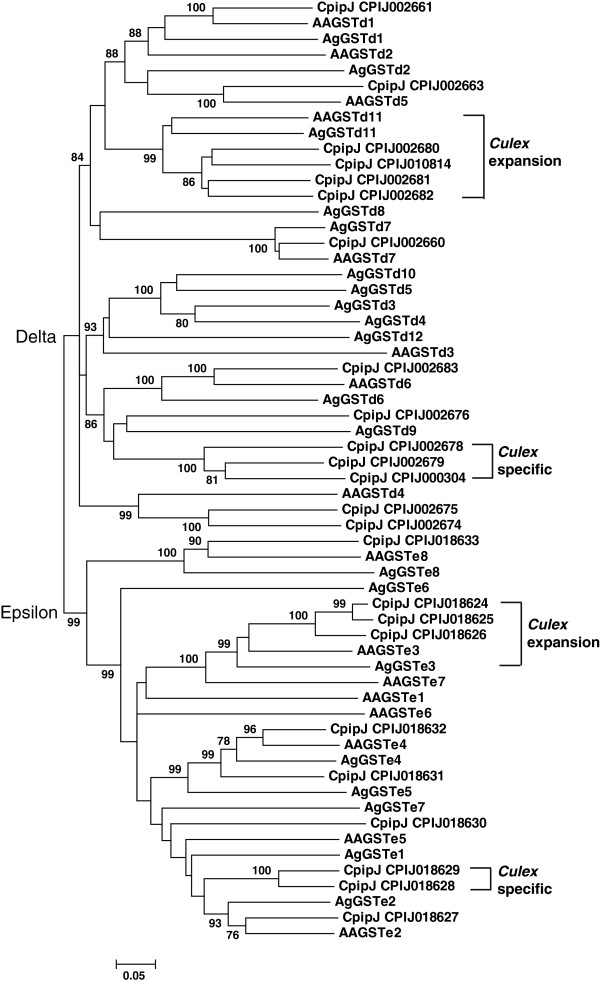
**Unrooted distance neighbor-joining tree showing the phylogeny of delta and epsilon glutathione S-transferases from the genome of *Culex pipiens quinquefasciatus *in relation to those from *Aedes aegypti *(initiated with ‘AA’) and *Anopheles gambiae *(initiated with ‘Ag’).** The percentage of bootstrap confidence values greater than 70% (1000 replicates) is shown at the nodes.

*C. p. quinquefasciatus* had 35 cytosolic GST genes belonging to the Delta, Epsilon, Omega, Sigma, and Theta clades (Table 1). The majority of the GSTs were represented by the Delta and Epsilon clades, which are insect-specific clades and contain the majority of the GSTs associated with detoxification in insecticides [22]. Many members of the Delta and Epsilon clades expanded locally in the *C. p. quinquefasciatus* genome, such as 12 Delta GSTs in supercontig 36 and 10 Epsilon GSTs in supercontig 1224 (Additional file 3). Delta 1, 6, and 7, and Epsilon 2, 4, and 8 showed 1:1:1 orthologies across the three mosquito genomes while Delta 11 and Epsilon 3 clades expanded on the *Culex* genome (Figure 7). Furthermore, *Culex* had specific Delta and Epsilon clades, which did not have clear orthologs in other two mosquito species (Figure 7). Unlike hymenopteran insects, where the Sigma class of GSTs expands and is thought to play an important role in protection against oxidative stress [9], only one Sigma GST gene was located in the genomes of dipteran insects. The Omega, Theta, and Zeta classes of GSTs are ubiquitously distributed in nature, but no Zeta GST has been identified in *C. p. quinquefasciatus*.

Why does *C. p. quinquefasciatus* have such an abundance of detoxification genes compared to other insect species? Several biological characteristics of mosquitoes may provide clues. The aquatic breeding sites of larvae and pupae contain numerous microorganisms, phenolic products of plant degradation, and pesticides. Adults feed on plant nectars and mammalian blood, which contain some harmful substances, such as heme and plant toxins. As viral pathogen vectors, mosquitoes have to deal with the generation of toxic endogenous compounds and reactive oxygen species during the immune response. But these cannot account for the gene expansion in *C. p. quinquefasciatus* compared to *Anopheles* and *Aedes* species. Perhaps its more polluted larval habitat and more diverse geographic range have exerted a greater selective pressure on *C. p. quinquefasciatus* so as to produce a larger repertoire of detoxification enzymes.

### DGE library sequencing and mapping to genome

**Figure 8:**
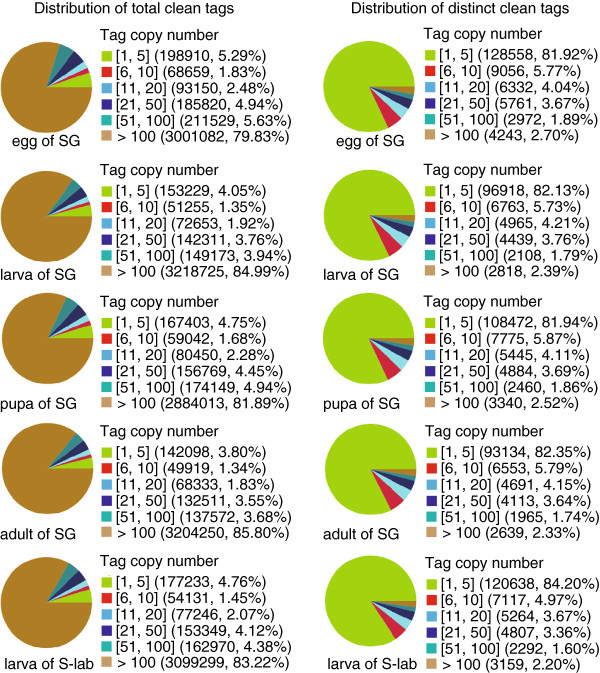
**Distribution of total clean tags and distinct clean tags over different tag abundance in each DGE library.** Numbers in square brackets indicate the range of copy numbers for a specific category of tags. Numbers in parentheses show the total number of tags in that category.

Using the DGE technique, which measures absolute, rather than relative, gene expression levels, the transcriptome was analyzed during the development of *C. p. quinquefasciatus*. For the five DGE libraries, 3.5 million to 3.8 million raw tags were generated (Table 2). The ratio of low quality reads was lower than 0.1% in all libraries except in that of the adult SG (Additional file 4). The number of distinct clean tags ranged from 113095 to 156922 (Table 2). The distribution of the total tags and distinct tags over different tag abundance showed similar patterns among the five libraries, indicating the normality of the DGE data (Figure 8). Highly expressed tags with copy numbers larger than 100 dominated in the distribution of the total clean tags, while tags with low expression and copy numbers smaller than 5 occupied the majority of the distinct clean tags (Figure 8). Pearson correlations between development stages ranged from 0.76 to 0.95, indicating uneven transcriptome divergence during mosquito development or the existence of lowly expressed genes not detected, while the Pearson correlation between the two third instar larva libraries of the SG and S-lab strains was relatively high (0.98), reflecting the reproducibility of DGE sequencing (Additional file 5). Among 20306 reference genes in VectorBase, a total of 13314 (65.6%) reference genes were mapped by unambiguous tags combining the five DGE libraries. Sequencing saturation analysis showed that the increase in the identified gene number nearly stopped when the number of reads reached 3 million (Additional file 6).

**Table 2:** DGE sequencing statistics

	**SG**								**S-lab**	
	**Egg**		**3rd Larva**		**Pupa**		**Adult**		**3rd Larva**	
	**N**	**%**	**N**	**%**	**N**	**%**	**N**	**%**	**N**	**%**
Total raw tag	3762501		3790500		3524500		3769500		3727501	
Total clean tag	3759150		3787346		3521826		3734683		3724228	
Distinct clean tag	156922		118011		132376		113095		143277	
Distinct tag mapping to gene	59206	37.7	42706	36.2	45666	34.5	41254	36.5	51264	35.8
Distinct unambiguous tag mapping to gene	48378	30.8	34772	29.5	37693	28.5	34449	30.5	39475	27.6
Unambiguous tag-mapped genes	10917	53.8	9808	48.3	10252	50.5	9759	48.1	10976	54.1
Distinct tag mapping to genome	48679	31.0	36533	31.0	40063	30.3	35062	31.0	47035	32.8
Total unknown tag	424236	11.3	397001	10.5	442991	12.6	415816	11.1	355720	9.6
Distinct unknown tag	49037	31.2	38772	32.9	46647	35.2	36779	32.5	44978	31.4

For each number (N) of tags or tag-mapped genes, the associated percentage (%) regarding the distinct clean tags (156922), the reference genes (20306), or total clean tags (3759150) is indicated.

### GO and KEGG pathway classification of the genes expressed in *C. p. quinquefasciatus*

**Figure 9:**
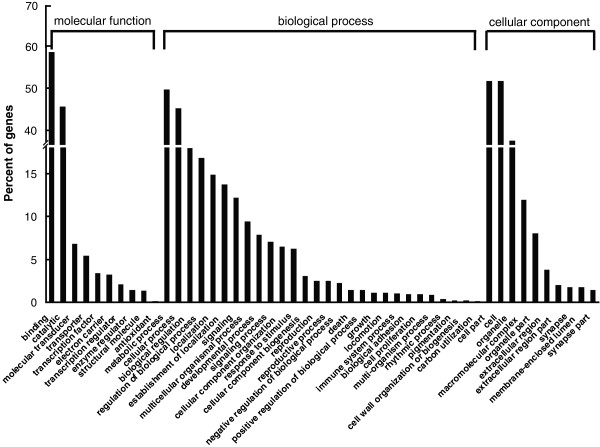
**Gene ontology classification of the genes expressed in *Culex pipiens quinquefasciatus.***

GO and KEGG pathway assignments were performed on the expressed genes to classify their functions and dissect the molecular events behind the expressed genes from the five DGE libraries. Of the 13314 genes, 2391 genes could be categorized into 48 GO function groups, among which binding, catalytic activity, metabolic process, cellular process, and cell part or cell were predominant categories. In contrast, few genes were classified into groups for antioxidant activity, rhythmic process, pigmentation, cell wall organization, and carbon utilization (Figure 9). A total of 1629 genes were mapped to 125 KEGG pathways. The most-represented pathways were metabolism (578 genes), microbial metabolism in diverse environments (101 genes), RNA transport (100 genes), spliceosome (95 genes), and protein processing in endoplasmic reticulum (95 genes).

### Life-stage specific detected genes

Comparing the four DGE libraries of the SG strain, 2666 genes were detected in only one library, and their functions and involvement largely diverged (Additional file 7). Among 302 detoxification genes, 225 (75%) were detected for expression in at least one DGE library (Table 1). About 25% of the genes that were not detected in any library were either not constitutively expressed in the life stages or were possibly untranscribed pseudogenes. Among expressed detoxification genes, 30% of the CCE genes, 25% of the P450 genes, and 72% of the GST genes were expressed in all life stages, indicating that they play a general housekeeping or detoxification function; some detoxification genes were developmental-stage specific (Table 3). Around 28% of the CCE genes were detected in only one stage, and two α-esterases, CpipJ CPIJ018232 and CpipJ CPIJ004752, were expressed at extremely high levels in larvae and pupae, respectively. Two insecticide-resistance-responsible esterases, CpipJ_CPIJ013917 for encoding esterase B and CpipJ CPIJ013918 for encoding esterase A, were expressed in all life stages, and their expression levels in larvae were higher than in adults. Only two GST genes showed stage-specific expression. Of the P450 genes, around 25% were detected in only one stage and most of them belonged to CYP4. Most strikingly, of 46 CYP325 genes, representing the widest expansion within the P450 family, 27 genes (59%) were detectable for expression and nearly half of the expressed genes (12 CYP325) were developmentally regulated. The larva and adult are feeding stages while the egg and pupae are non-feeding. Hence enzymes expressed specifically or highly in larva or adult are important in digestion and detoxification of dietary component whereas those in egg or pupae are vital for detoxification of metamorphosis byproducts or synthesis of specific hormones.

**Table 3:** Developmental-stage specifically expressed genes of carboxylcholinesterase (CCE), glutathione-S-transferase (GST), and cytochrome P450 monooxygenase (P450)

**Family**	**Stage**	**Gene**	**Class**	**TPM**
CCE	Egg	CpipJ_CPIJ007135	F	0.27
		CpipJ_CPIJ016026	B	0.27
		CpipJ_CPIJ009768	M	0.53
		CpipJ_CPIJ013027	F	0.53
	Larva	CpipJ_CPIJ016341	B	0.26
		CpipJ_CPIJ016682	G	2.00
		CpipJ_CPIJ018231	B	5.81
	Pupa	CpipJ_CPIJ018232	B	32.74
		CpipJ_CPIJ013679	B	0.57
		CpipJ_CPIJ013175	F	13.63
	Adult	CpipJ_CPIJ004752	B	118.69
		CpipJ_CPIJ016681	G	0.54
		CpipJ_CPIJ005122	G	5.62
GST	Larva	CpipJ_CPIJ002674	Delta	3.96
		CpipJ_CPIJ014051	Theta	7.66
P450	Egg	CpipJ_CPIJ019395	CYP4C52 v2	0.27
		CpipJ_CPIJ009478	CYP4D42 v1	0.27
		CpipJ_CPIJ017198	CYP325BF1	0.27
		CpipJ_CPIJ003361	CYP6BY2	0.80
		CpipJ_CPIJ017244	CYP304B5	2.00
	Larva	CpipJ_CPIJ009474	CYP4D40	0.26
		CpipJ_CPIJ010228	CYP12F12 v1	0.26
		CpipJ_CPIJ015428	CYP6Z10	0.26
		CpipJ_CPIJ001755	CYP4J19	0.26
		CpipJ_CPIJ014220	CYP9M12	0.53
		CpipJ_CPIJ014219	CYP9M11P	1.06
		CpipJ_CPIJ014730	CYP325AA2	1.06
		CpipJ_CPIJ007188	CYP4H30	1.32
		CpipJ_CPIJ007091	CYP325Y6 v2	1.58
		CpipJ_CPIJ011127	CYP4H34	1.85
		CpipJ_CPIJ010075	CYP4H35	2.11
		CpipJ_CPIJ003376	CYP6BY4	2.64
		CpipJ_CPIJ007089	CYP325Y4	4.22
		CpipJ_CPIJ015223	CYP6F4	6.6
		CpipJ_CPIJ010858	CYP6F1	47.0
		CpipJ_CPIJ014942	CYP305A10	1.00
	Pupa	CpipJ_CPIJ001754	CYP4J6	0.28
		CpipJ_CPIJ007095	CYP325Y9	0.28
		CpipJ_CPIJ005685	CYP325BB2	0.28
		CpipJ_CPIJ010810	CYP325BC2	0.28
		CpipJ_CPIJ004410	CYP6Z11	0.85
		CpipJ_CPIJ009477	CYP4D19	1.42
	Adult	CpipJ_CPIJ019587	CYP6Z14	0.27
		CpipJ_CPIJ006951	CYP325BG2P	0.27
		CpipJ_CPIJ010542	CYP9J38	0.27
		CpipJ_CPIJ003377	CYP6BY5	0.54
		CpipJ_CPIJ015960	CYP325BD1	1.87
		CpipJ_CPIJ011837	CYP325V2	2.68
		CpipJ_CPIJ019586	CYP6Z13P	2.68
		CpipJ_CPIJ006950	CYP325BG1	3.21
		CpipJ_CPIJ010203	CYP9AM1	5.36
		CpipJ_CPIJ015957	CYP325G4	51.68
		CpipJ_CPIJ009471	CYP4AR4	2.00

TPM, number of transcripts per million clean tags.

For gene expansion clusters of detoxification genes, the expression profiles were different among the members. For example, among the six members of one expanded α-esterase cluster (Figure 2), CpipJ_CPIJ007825 was detected for expression in pupae and adults while the other five members were not detected in any stage. For the seven members of another expanded α-esterase cluster (Figure 2), CpipJ_CPIJ016025, CpipJ_CPIJ005694, and CpipJ_CPIJ008749 were not detected for expression; CpipJ_CPIJ000049 and CpipJ_CPIJ000051 were expressed in pupae and adults, CpipJ_CPIJ000050 in larvae and pupae, and CpipJ_CPIJ016026 only in eggs. Similar phenomenon was observed in the three expansion clusters of juvenile hormone esterases (Figure 3). For the five members of cluster, only CpipJ_CPIJ013027 was detected for expression and in eggs. No expression was found in the three members of cluster. For the four members of cluster, three were detected for expression: CpipJ_CPIJ016681 and CpipJ_CPIJ016682 in adults, and CpipJ_CPIJ017763 in larvae and adults. The different expression patterns of these duplicated detoxification genes are probably indicative of their subfunctionalization or retrogression as pseudogenes.

### Differentially expressed genes between parathion resistant and susceptible larvae

When the third instar DGE library of the parathion-resistant strain SG was compared with the same stage in the susceptible strain S-lab, a total of 1511 genes showed different expression levels, among which 619 genes had up-regulated expression levels in the SG strain (Additional file 8). The most prominent GO functions of these up-regulated genes were endopeptidase or serine-type peptidase activity, such as genes encoding trypsin, chymotrypsin, mast cell protease 2, urokinase-type plasminogen activator, and elastase. However, not all of the differentially expressed genes are responsible for parathion resistance because comparison strains were not selected from the same panmictic population such that genetic background differences could be ruled out.

A total of 15 detoxification genes were expressed at higher levels in the SG strain, including 2 CCEs, 6 GSTs, and 7 P450s (Table 4). The expression of the known esterase B gene, CpipJ CPIJ013917, increased 16-fold in the resistant strain, while the esterase A gene, CpipJ CPIJ013918, did not show differential expression between the two strains. Three Epsilon GSTs had prominent involvement in resistance, as previously reported in DDT- and OP-resistant mosquitoes and houseflies [22,23]. Seven P450 genes with elevated expression in the resistant strain belonged to either the CYP9 or CYP6 groups, which are usually implicated in pyrethroid resistance in mosquitoes and other species [18]. P450 monooxygenases are relatively less commonly involved than carboxylesterases in resistance to OP insecticides, although evidence shows that CYP6A1 from *Musca domestica* and CYP6A2 from *Drosophila* are capable of metabolizing diazinon [24,25]. Unless direct evidence demonstrates the detoxification or sequestration of insecticide compounds by P450s, the conclusion that the 7 up-regulated P450 genes are involved in OP resistance is too early to draw. Most of the 15 detoxification genes were expressed in all life stages, while 6 were expressed only in certain stages, especially Theta GST (CpipJ CPIJ014051), CYP9J34 (CpipJ CPIJ010546), and CYP6F1 (CpipJ CPIJ010858), which were not found in adults. The different responses of detoxification genes may account for the different resistance levels between the larval and adult stage in some circumstances.

**Table 4:** Detoxification genes up-regulated in parathion resistant larvae of the SG strain

**Gene family**	**Classification**	**Gene number**	**log**_**2**_Ratio^*^	**Expression stage**
CCE	α esterases	CpipJ_CPIJ013917	4.4	All
	JH esterases	CpipJ_CPIJ002073	1.7	All
GST	Epsilon	CpipJ_CPIJ018629	8.7	All
	Epsilon	CpipJ_CPIJ018632	5.0	All
	Epsilon	CpipJ_CPIJ018627	3.5	All
	Theta	CpipJ_CPIJ014051	2.2	Larva
	Delta	CpipJ_CPIJ002675	1.9	Egg, larva, adult
	Others	CpipJ_CPIJ014694	1.1	All
P450	CYP9J34	CpipJ_CPIJ010546	4.0	Larva, pupa
	CYP9J40	CpipJ_CPIJ010543	3.3	Larva, pupa, adult
	CYP6AG11	CpipJ_CPIJ002537	3.0	All
	CYP6BZ2	CpipJ_CPIJ005956	2.6	All
	CYP9AE1	CpipJ_CPIJ000655	2.0	All
	CYP6F1	CpipJ_CPIJ010858	1.3	Larva
	CYP9AL1	CpipJ_CPIJ012470	1.1	Larva, adult

*Ratio, TPM of SG/TPM of Slab. TPM, number of transcripts per million clean tags.

## Conclusion

*C. p. quinquefasciatus* is an important vector that transmits human diseases different from those by *An. gambiae* and *Ae. aegypti*. The lack of transcriptomic data available for this species has hampered characterization of the molecular mechanisms underlying the different biological characters of the three major mosquito vectors. The five DGE libraries described in the present study represent a dramatic expansion of the existing transcriptomic sequence available for *C. p. quinquefasciatus*. This expansion will facilitate the investigation of the fundamental biology of *C. p. quinquefasciatus* and its pathogenic interactions. In addition, the results of the present study provide new insights into the functions and evolution of the three detoxification gene families of mosquitoes. A larger number of detoxification genes were identified on the genome of *C. p. quinquefasciatus* compared with three other dipteran insect genomes, representing the widest gene expansion sequenced thus far. Comparative genomic analysis suggested that gene expansion mainly occurs in α-esterases, juvenile hormone esterases, and P450 CYP325. Some detoxification genes were expressed in all developmental stages, while some were developmentally regulated. The expression profiles were different among the members of gene expansion clusters, probably indicative of their subfunctionalization or retrogression as pseudogenes. Fifteen detoxification genes showed the potential to take part in the parathion resistance of *Culex*, including unexpected P450 genes.

## Methods

### Mosquito strains

Mosquito strains of *C. p. quinquefasciatus* used included S-lab, which was OP-susceptible and reared at the laboratory without any contact with insecticides for many years [26] and Shengui (SG), a field population collected in Foshan, Guangdong Province, in 2007 and constantly treated with parathion at the laboratory. The parathion-resistance of SG was 115-fold that of S-lab before use in the DGE analysis. The mosquitoes were maintained at 26°C ± 1°C and a long-day photoperiod (14 h light/10 h darkness cycle). Fifty egg rafts, forty third instar larvae, forty pupae, and forty adults (twenty females and twenty males) of SG and forty third instar larvae of S-lab were collected and frozen at −80°C for further analysis.

### Identification and phylogenetic classification of detoxification genes

Sequences encoding GSTs, P450s, and CCEs were identified from the protein set of the *C. p. quinquefasciatus* whole genome sequencing database at the Broad Institute ( http://www.broadinstitute.org/annotation/genome/culex_pipiens) using the HMMER program ( http://hmmer.janelia.org/) with the protein domains for CCEs (PF00135), GSTs (PF00043 and PF02798), and P450s (PF00067) as described in the Pfam database. A significance value of 1e^-10^ was used in the searches for CCEs and P450 and 2e^-2^ for GSTs. Community annotations and VectorBase were referred to verify the searches [27]. Those candidate genes not supported by the community annotations as CCEs, P450 or GST were not accounted. P450s were named by the P450 nomenclature committee ( http://drnelson.uthsc.edu/CytochromeP450.html). Known detoxification genes from *An. gambiae*, *D. melanogaster*[28], and *Ae. aegypti*[17] were used as references for the phylogenetic classification of the detoxification genes from *C. p. quinquefasciatus*. Protein sequences were aligned with ClustalW2 at EMBL-EBI ( http://www.ebi.ac.uk/Tools/msa/clustalw2). Unrooted distance neighbor-joining trees showing the phylogeny of detoxification gene families were constructed using the pairwise deletion and p-distance functions of Mega 4.0 software. Bootstrap analysis (1000 replicates) was applied to evaluate the internal support of the tree topology.

### Pipeline of DGE

Six micrograms of total RNA from each of the above five mosquito samples were isolated using TRIzol reagent (Invitrogen, Carlsbad, CA, USA) according to the manufacturer’s instructions. Tag library preparation was performed with an Illumina Gene Expression Sample Prep Kit. The raw data (tag sequences and counts) were deposited in the NCBI Sequence Read Archive (SRA) database under submission number SRA049959.

### Pipeline of bioinformatics analysis on DGE

Sequencing-received raw image data were transformed by base calling into raw sequence data. Clean tags were obtained after raw sequences were filtered to remove adaptor sequences, empty tags, low quality tags, tags that were too long or too short, and tags with a copy number of 1. The distribution of clean tags was used to evaluate the normality of the whole data. Saturation analysis was performed to determine whether or not the number of detected genes continues to increase when the sequencing amount increases. Pearson correlation analysis of two parallel libraries was performed to evaluate the reliability and operational stability of the experimental results. All clean tags were mapped to *C. p. quinquefasciatus* whole genome reference sequences and allowed no more than 1 nucleotide mismatch. The number of unambiguous clean tags for each gene was calculated and then normalized to TPM (number of transcripts per million clean tags). When the expression of a gene was not detected, TPM was set to 0.01.

A rigorous custom written algorithm using the method described by Audic et al. [29] was developed to identify differentially expressed genes between two samples. The p value corresponded to the differential gene expression test. False discovery rate (FDR) was used to determine the p value threshold in multiple tests and analyses [30]. FDR ≤ 0.001 and the absolute value of log_2_Ratio ≥ 1 were used as thresholds to judge the significance of the gene expression difference.

Unigenes matched by clean tags were assigned to Gene Ontology (GO) terms using Blast2GO and canonical pathways in KEGG (Kyoto Encyclopedia of Genes and Genomes). GO or pathway enrichment analysis of the differentially expressed genes was performed based on the algorithm presented by GOstat [31]. The difference between the differentially expressed gene group and the whole gene expression background was represented by a p value, which was approximated by a chi-square test. The Fisher exact test was used when any expected count value was below 5, which will result in inaccurate chi-square test results. Benjamini multiple-testing correction of the p value was done by FDR.

## Competing interests

The authors declare that they have no competing interests.

## Authors’ contributions

LY and NC prepared the RNA, performed the phylogenetic analysis of the detoxification genes, and drafted the manuscript. PY and FJ performed the GO and pathway enrichment analysis of differentially expressed genes. EM and CQ conceived and designed the experiment. FC designed the experiment, performed the phylogenetic analysis of the detoxification genes, and wrote the manuscript. All authors read and approved the final manuscript.

## Supplementary Material

Additional file 1 **Summary of the carboxylcholinesterase genes of*Culex pipiens quinquefasciatus.*** TPM, number of transcripts per million clean tags. When the expression of a gene was not detected, TPM was set to 0.01. Click here for file

Additional file 2 **Summary of the cytochrome P450 monooxygenase genes of*Culex pipiens quinquefasciatus.*** TPM, see Additional file 1. Click here for file

Additional file 3 **Summary of the glutathione S-transferase genes of*Culex pipiens quinquefasciatus.*** TPM, see Additional file 1. Click here for file

Additional file 4 Distribution of the total tags in each DGE library. Click here for file

Additional file 5 **Pearson correlation analysis of the DGE libraries.** Dots in the figures indicate individual tag entities. TPM (Transcripts Per Million clean tags) indicates the number of transcript copies in every 1 million clean tags. A–F, correlation between the four developmental stages of the SG strain; G, correlation between third instar larvae of the SG and S-lab strains. Click here for file

Additional file 6 Relationship between the number of identified genes and sequencing amount. Click here for file

Additional file 7 Enriched GO function groups^*^and KEGG pathways involved by genes specifically expressing in various life stages ofCulex pipiens quinquefasciatus.^*^GO function groups include three main categories: biological process (BP), molecular function (MF), and cellular component (CC). ^†^The p value represented the difference between the specifically expressed gene group and the total 13,314 gene group approximated by chi-square test. Fisher exact test is used when any expected count value is below 5. ^‡^Benjamini is the multiple-testing correction of the p value by FDR. Click here for file

Additional file 8 **Differentially expressed genes between the parathion-resistant and susceptible larvae of*Culex pipiens quinquefasciatus.*** TPM, see Additional file 1. Click here for file
