# Supplementary figures and images for: Transcriptomic and phylogenetic analysis of Culex pipiens quinquefasciatus for three detoxification gene families
Source: BMC Genomics. 2012 Nov 10;13:609. doi: 10.1186/1471-2164-13-609 (PMC3505183; doi:10.1186/1471-2164-13-609)

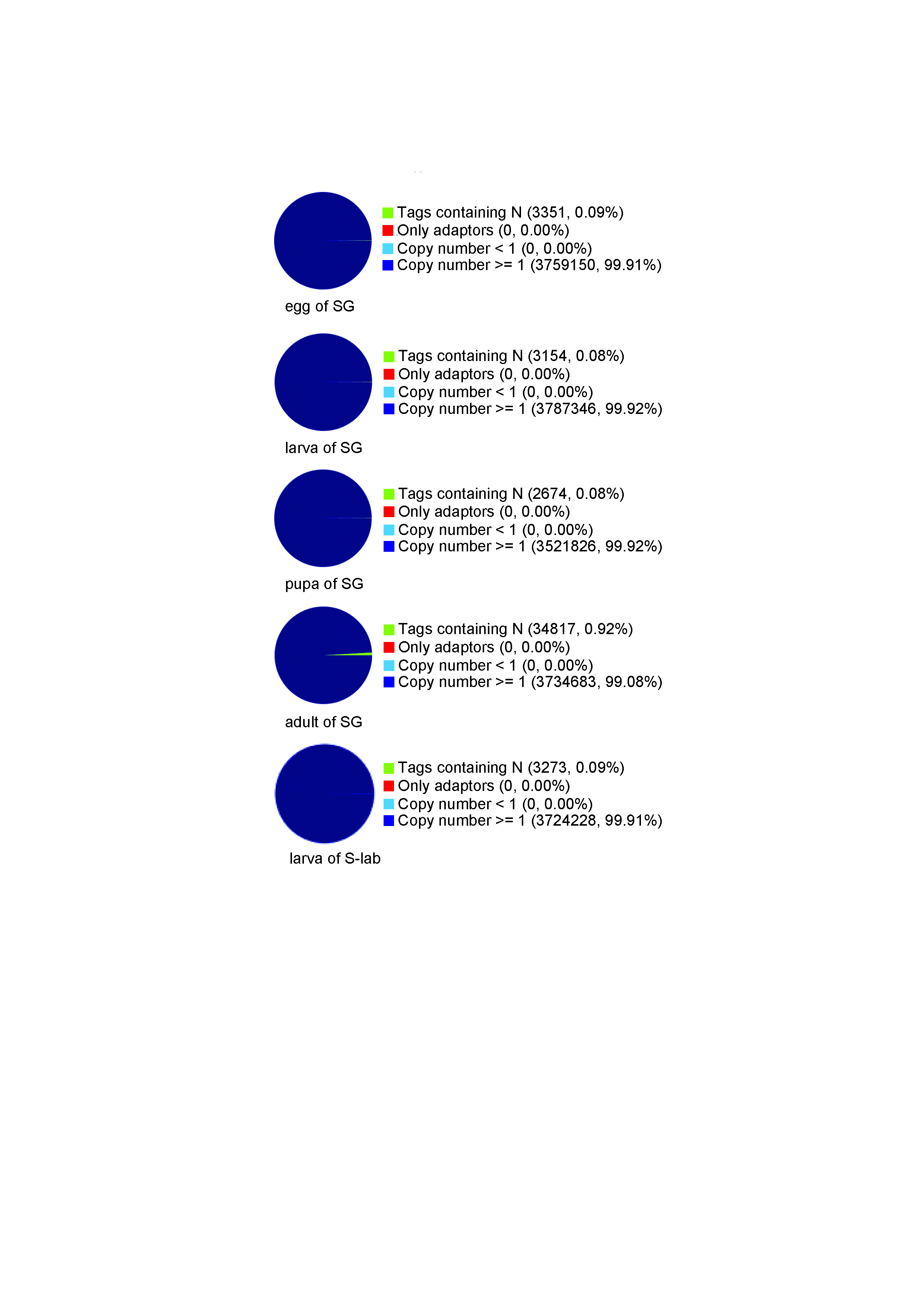

Supplement: Additional file 4 — Distribution of the total tags in each DGE library. [file 1471-2164-13-609-S4.tiff]

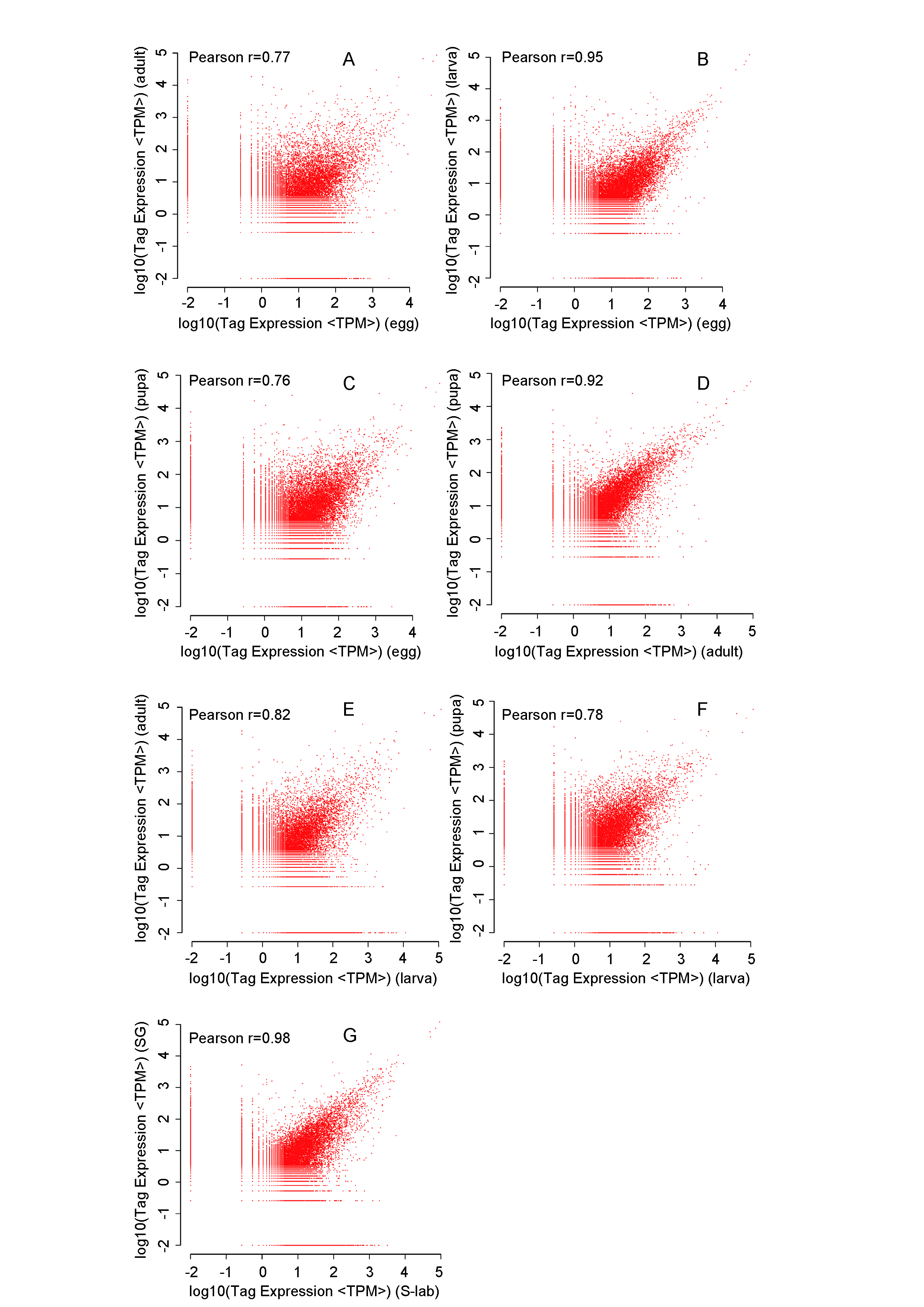

Supplement: Additional file 5 — Pearson correlation analysis of the DGE libraries. Dots in the figures indicate individual tag entities. TPM (Transcripts Per Million clean tags) indicates the number of transcript copies in every 1 million clean tags. A–F, correlation between the four developmental stages of the SG strain; G, correlation between third instar larvae of the SG and S-lab strains. [file 1471-2164-13-609-S5.tiff]
