# Supplementary material for: Transcriptomic and phylogenetic analysis of Culex pipiens quinquefasciatus for three detoxification gene families
Source: BMC Genomics. 2012 Nov 10;13:609. doi: 10.1186/1471-2164-13-609 (PMC3505183; doi:10.1186/1471-2164-13-609)
Supplement: Additional file 7 — Enriched GO function groups*and KEGG pathways involved by genes specifically expressing in various life stages ofCulex pipiens quinquefasciatus.*GO function groups include three main categories: biological process (BP), molecular function (MF), and cellular component (CC). †The p value represented the difference between the specifically expressed gene group and the total 13,314 gene group approximated by chi-square test. Fisher exact test is used when any expected count value is below 5. ‡Benjamini is the multiple-testing correction of the p value by FDR. [file 1471-2164-13-609-S7.doc]

**Additional file 7 Enriched GO function groups^*^ and KEGG pathways involved by genes specifically expressing in various life stages of *Culex pipiens* *quinquefasciatus***

| GO or KEGG ID | GO or KEGG term | p value^†^ | Benjamini^‡^ |
| --- | --- | --- | --- |
| Egg specific (1076 genes) | |  |  |
| GO:0004871 | Signal transducer activity (MF) | 1.51E–05 | 2.16E–03 |
| GO:0005549 | Odorant binding (MF) | 8.44E–05 | 6.03E–03 |
| GO:0023033 | Signaling pathway (BP) | 4.33E–04 | 0.021 |
| KEGG:04340 | Hedgehog signaling pathway | 1.86E–03 | 0.099 |
| KEGG:04310 | Wnt signaling pathway | 0.021 | 0.35 |
|  |  |  |  |
| Larva specific (545 genes) | |  |  |
| GO:0008233 | Peptidase activity (MF) | 3.41E–06 | 3.45E–04 |
| GO:0006508 | Proteolysis (BP) | 4.79E–05 | 4.16E–03 |
| KEGG:00603 | Glycosphingolipid biosynthesis | 4.39E–03 | 0.079 |
| KEGG:00230 | Purine metabolism | 0.012 | 0.11 |
|  |  |  |  |
| Pupa specific (465 genes) | |  |  |
| GO:0008354 | Germ cell migration (BP) | 9.37E–03 | 0.31 |
| GO:0031110 | Microtubule polymerization or depolymerization (BP) | 0.014 | 0.31 |
| KEGG:04144 | Endocytosis | 0.013 | 0.37 |
| KEGG:04310 | Wnt signaling pathway | 0.040 | 0.37 |
|  |  |  |  |
| Adult specific (580 genes) | |  |  |
| GO:0030001 | Metal ion transport (BP) | 0.026 | 0.55 |
| GO:0031420 | Alkali metal ion binding (MF) | 0.027 | 0.55 |
| KEGG:00982 | Drug metabolism — cytochrome P450 | 5.82E–03 | 0.15 |
| KEGG:00330 | Arginine and proline metabolism | 0.033 | 0.21 |
| KEGG:00564 | Glycerophospholipid metabolism | 0.044 | 0.21 |

^*^GO function groups include three main categories: biological process (BP), molecular function (MF), and cellular component (CC).

^†^The p value represented the difference between the specifically expressed gene group and the total 13,314 gene group approximated by chi-square test. Fisher exact test is used when any expected count value is below 5.

^‡^Benjamini is the multiple-testing correction of the p value by FDR.
